# Demographic, Social and Health-Related Variables that Predict Normal-Weight Preschool Children Having Overweight or Obesity When Entering Primary Education in Chile

**DOI:** 10.3390/nu11061277

**Published:** 2019-06-05

**Authors:** Juliana Kain, Bárbara Leyton, Louise Baur, Mariana Lira, Camila Corvalán

**Affiliations:** 1 Instituto de Nutrición y Tecnología de los Alimentos (INTA), Universidad de Chile, El Líbano 5524, Macul, Santiago, Chile; bleyton@inta.uchile.cl (B.L.); ccorvalan@inta.uchile.cl (C.C.); 2 Sydney Medical School, University of Sydney, Sydney NSW 2006, Australia; Louise.baur@health.nsw.gov.au; 3 Junta Nacional de Auxilio Escolar y Becas (JUNAEB), Antonio Varas 153 Santiago, Chile; mariana.lira@junaeb.cl

**Keywords:** preschool children, overweight, obesity, predictive variables, Chile

## Abstract

We determined which variables are predictive of normal-weight (N) Chilean 4-year-olds developing overweight/obesity when entering primary school. This study used national data of preschoolers (PK, age 4) in 2011 through 2015, and the same children in the first grade (1st G, age 6) in 2013 through 2017. We formed longitudinal cohorts considering PK as the baseline and 1st G as the follow-up and included anthropometric, socio-demographic, and health variables in PK and anthropometry in the 1st G. We report the percentage N who remained N at follow-up (N-N) or gained excessive weight (N-OW) and (N-OB), by sex. We ran univariate logistic regressions to determine for each variable, its association with gaining excessive weight (N-OW + OB), incorporating significant variables (*p* < 0.001) in multivariate logistic regression. A total of 483,509 (251,150 girls) of PK had anthropometry in the 1st G. In PK, 22% of the children were obese; in the 1st G (24.8% and 19.7% in boys and girls, respectively). Of normal-weight children, 30% developed OW + OB. The predictive variables were: Being born macrosomic, attending a very vulnerable school, being indigenous, the mother’s low schooling, and the child being cared for by the grandmother after school. In this study, the factors predicting that normal-weight preschoolers gain excessive weight gain in a short period of time are mostly related to poverty. Prevention should focus on this population.

## 1. Introduction

There is a large body of evidence showing that excessive weight gain in early childhood is associated with obesity in adolescence and adulthood [1,2,3,4]. The age of onset of obesity is important. For example, in a German study which included more than 50,000 children with anthropometric data from birth to 17 years of age, 90% of those with obesity at three years of age were affected by being overweight or obese at 14 years [5]. Among adolescents with obesity, the highest increments in weight gain had occurred between two and six years of age. Barraclough et al. [6] found that an early excessive weight gain from two years of age that persisted through adolescence, resulted in an increased cardio-metabolic risk independent of the achieved body mass index (BMI). Such findings reflect the importance of prioritizing measures to prevent excessive weight during the preschool years.

Chile has one of the highest obesity rates in the world: Nationally representative data from 2018 show that the prevalence of obesity is 23.7%, 24.6% and 24.4% in four, five and six-year-old children, respectively. In addition, around 26% in each age group is overweight, so half of the children between four and six years of age are either overweight (OW) or present obesity (OB) [7].

The risk factors for developing early childhood obesity include both prenatal and postnatal factors. The prenatal factors reported more often are maternal pre-pregnancy obesity and diabetes, excessive gestational weight gain and smoking during pregnancy, while postnatal ones include high birth weight and length, reduced breastfeeding, inappropriate infant feeding practices, inadequate sleep quality and duration, and several socioeconomic aspects, such as ethnicity and family income [8,9].

In the Growth and Obesity Chilean study, we described growth patterns of ~1000 children, showing that all periods until first grade are relevant in terms of the obesity incidence [10,11]. We have also observed that prenatal and early life factors are critical for obesity development [12,13]. However, information on obesity emergence during the preschool years and its determinants on a larger sample are still lacking; this is relevant for countries facing advanced stages of nutritional transition such as Chile, and can highlight important areas for preventive actions in these settings. Therefore, the aim of this study was to describe in a representative sample of Chilean preschool children attending public schools, the demographic, social, and health-related determinants, which predict that healthy weight (normal-weight) four-year-old children develop overweight or obesity at six years of age (the beginning of school education).

## 2. Methods

### 2.1. Data Sets

We obtained the databases from the Junta Nacional de Auxilio Escolar y Becas, an agency which belongs to the Chilean Ministry of Education. It is in charge of collecting the data from the annual census of anthropometric, socio-demographic and health variables of preschool, prekindergarten or four-year-old students (PK) and first-grade students, aged six years old, attending kindergartens and/or public schools in the country. This census covers 76% and 68% of all preschool and first-grade children in the country, respectively.

The databases include anonymized socioeconomic and health-related variables on individual children who attended prekindergarten in 2011, 2012, 2013, 2014 and 2015 and same children in first grade in 2013, 2014, 2015, 2016 and 2017. Most variables originated from a survey administered annually by the head teacher to the mother/guardian in the first semester at every kindergarten and school [14]. The exceptions were for weight and height—which were measured by a trained physical education teacher who followed a protocol—and birth weight and length, which we obtained from the Ministry of Health.

### 2.2. Participants

We formed a database linking the data of the same children when they were in prekindergarten (PK; baseline) and in first grade (1st G; follow-up). We calculated the age and sex-standardized body mass index (BMI Z) for all the children at baseline and follow-up, using the AnthroPlus software of the World Health Organization (WHO).

The children included in the final analyses were those who had plausible anthropometric data at baseline and at follow-up, and information on all variables at baseline, while excluded ones were the children for whom we did not have these data. We considered the anthropometric data plausible if the date of measurement was credible and if their BMI Z was between −5 and 5 [15], although there were no children with BMI Z < −3. To determine if the children included in the study were different from those excluded, we compared if there were differences in the mean age and percentage of children with overweight plus obesity at baseline, using the test of proportions. A *p*-value < 0.05 was considered significant.

### 2.3. Variables

For this study we considered the following variables collected by the survey when children attended PK [14]: The age, sex, weight, height, membership of an indigenous group, frequency of health check-ups, daycare attendance at two and/or three years of age, breastfeeding duration, person in charge of the child after school, the maternal and paternal educational level and type of job, the school´s vulnerability index (percentage of children requiring free school meals was used as a proxy), urban or rural residence. In the first grade, we obtained weight and height data for the same children.

### 2.4. Statistical Analyses

For each of the variables, we verified implausible values and assessed their distribution. All variables were distributed normally and, therefore, they are presented as the mean and standard deviation. In order to assess risk factors for obesity development, all variables were categorized based on standard cutoff values. We describe the variables listed above in all the PK children (2011–2015) as percentages, separately for boys and girls and determined if there were significant differences by sex, using the test of proportions. A *p*-value < 0.05 was considered as significant.

We determined the nutritional status at baseline using the WHO Reference 2017 [15], that is: Low weight (BMI Z ≤ −1); normal weight (BMI Z > −1 and ≤1; overweight (BMI Z >1 and ≤2) and obese (BMI Z > 2) and the mean BMI Z, by sex. Because of the small percentage of children with low weight, these were included in the normal-weight group.

Three categories were formed to determine the percentage of normal-weight children at baseline who remained in the same weight category at follow-up (N-N) and the percentage at follow-up who moved into the overweight (N-OW) or obesity (N-OB) categories. Because the percentage of normal-weight children who became overweight or developed obesity (N-OW + N-OB) was similar each year, for the comparative analyses of predictive variables, a single category was formed (N-OW + OB).

In order to determine which variables were associated with the outcome (N-OW + OB) we ran simple logistic regressions with each one of them, stratifying by sex, where a *p*-value < 0.05 was considered significant. In the case of the birthweight, we determined this association with two categories: <2500 and ≥4000 g. We then ran multiple logistic regression models that included all variables that resulted in significance in the univariate analyses, by sex. All analyses were conducted with Stata 15.1 (Stata Corp, College Station, TX, USA).

### 2.5. Ethics

This study was reviewed and approved by INTA’s Ethics Committee of the University of Chile (Approval N° 7/2019).

## 3. Results

**Figure 1:**
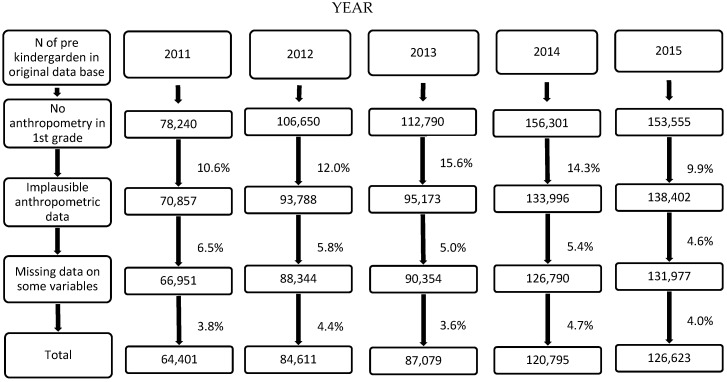
Population flow chart.

The original databases included 607,536 children in PK from 2011 to 2015 (Figure 1). Of these, between 10–15% did not have anthropometry data in 1st grade. In addition, around 5.5% had to be eliminated for implausible data on weight and/or height at baseline and/or follow-up, plus an additional 4% for not having data on all the variables at baseline. Thus, the final population of children considered for the analyses (baseline and follow-up) was 483,509 (251,140 girls) fluctuating from 64,401 in 2011 to 126,623 in 2015.

No differences were observed between the included and excluded individuals in relation to the percentage of children being overweight and presenting obesity at baseline (*p* = 0.31). However, in spite of the very small difference in the average age (0.4 months) between included (54.83 months) and excluded (54.42 months), the difference was significant.

Table 1 shows some of the socioeconomic and health-related characteristics of the population of PK, by sex. Previous attendance at daycare centers was moderate (44% in both sexes), about half of the children attended very vulnerable schools, 43% of mothers did not hold a formal job, 22% of mothers and fathers only had primary education, while 25% of fathers were unemployed. More than a quarter of the children were cared for by a grandmother or another relative after school (80% by a grandmother). The proportion of boys whose birth weight was ≥4000 g (macrosomic) was significantly higher than girls, 10.8% and 6.8%, respectively (*p* < 0.05).

Table 2 and Table 3 show the mean prevalence rates for overweight and obese children, as well as the mean (SD) BMI Z in prekindergarten (PK) children at baseline and at follow-up two years later (in the 1st grade), in boys and girls, respectively. In boys, the mean prevalence of overweight in PK and the 1st grade within 2011–2015 was 27.9% and 26.8%, respectively, while that for obesity was 22.1% and 24.8%, with minimum variation between years. The mean BMI Z in PK and first-grade children for the period was in the range of overweight. Girls had similar prevalence rates for overweight in PK and the 1st grade, however, the obesity prevalence was around 3 percentage points lower than in boys and also had less variation between years. The mean BMI Z was slightly lower than in boys.

**Figure 2:**
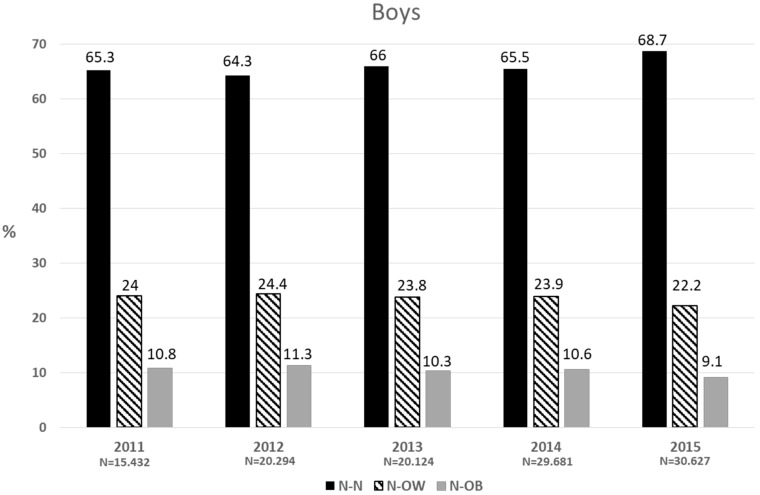
Two-year change (percentage) in the nutritional status of normal-weight prekindergarten children.

**Figure 3:**
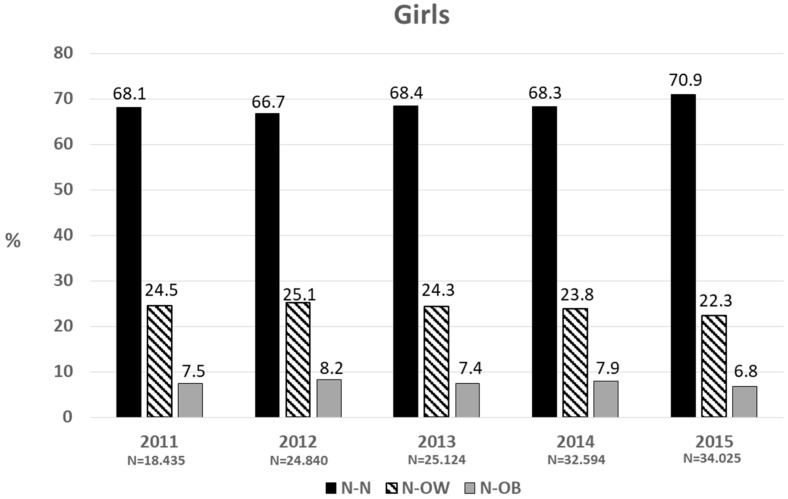
Two-year change (in percentage) in the nutritional status of normal-weight prekindergarten children.

Figure 2 and Figure 3 show the proportion of normal-weight children in prekindergarten who progressed either to normal weight status, overweight or obesity by first grade, each year, in boys and girls, respectively. Of the boys who were normal weight in prekindergarten, by the time they were in first grade, around two-thirds remained in the normal weight category (N-N), one-quarter became overweight (N-OW) and one-tenth developed obesity (N-OB). In girls, the proportion of N-N was around 2.5 percentage points higher than in boys, while the percentage of N-OB was lower by the same proportion. The average percentage of N-OW + N-OB for the five years was 34.1% and 31.6% in boys and girls, respectively. In the same period, a significantly lower proportion of overweight preschool children became normal-weight (15.2% for the total sample, of which 11.2% corresponded to overweight preschoolers) (not shown).

In order to run the regression models, we pooled together the N-OW and N-OB groups and formed one category (N-OW + OB) to be compared to N-N. We ran a univariate logistic regression with each variable separately for each sex. The following variables were associated with normal-weight children being in the overweight or obesity category two years later: Belonging to an indigenous ethnic group, living in rural areas, mothers and/or fathers with only primary or secondary education (just for girls), unemployed fathers, the child looked after by grandmother or another relative, a birth weight ≥ 4000 g and the child attending a more vulnerable school. Low birth weight resulted as a protective variable to gaining excessive weight during the period, (OR 0.76 (0.72–0.81)) for both sexes (not shown).

We then ran the final logistic regression model separately for each sex (Table 4). For boys being indigenous, having childcare provided by a grandmother or another relative after school, having a high birth weight, attending a very vulnerable school, and having a mother with only primary education were the variables more strongly associated with developing OW or OB. For girls, additional factors included mothers with less than 12 years of schooling and fathers holding a sporadic job. We can observe that the variables urban/rural residence and father´s educational level were excluded in the final multivariate regression model (Table 4).

## 4. Discussion

The main finding of this study shows that normal-weight four-year-old children can gain excessive weight in a short period of time. Within only two years, one-quarter of the children developed overweight, with around 10.5% and 7.5% of normal-weight boys and girls, respectively, developing obesity by first grade. The predictive variables that were associated with excessive weight gain during this period were similar for boys and girls: A high birth weight, the child looked after school by a grandmother, and poverty-related factors, such as attending a very vulnerable school, being indigenous, and mother having only primary education.

There is an upward trend in the prevalence of obesity among Chilean children. In 2012, it was 21.9% and 23.6% among four and six-year-old children, respectively. While in 2018, it was 23.7% and 24.4%, respectively [7]. While this information is widely disseminated, the fact that 30% of normal-weight preschool children can gain excessive weight in just two years, from prekindergarten to first grade, was not known. Excessive weight gain in that age group during the period analyzed was similar, except for 2015, in which there was a smaller proportion of both N-OW and N-OB. This coincides with the slightly lower prevalence of obesity observed in 2015 among children attending first grade in Chilean public schools. This result cannot be considered as a downward trend, but just a small fluctuation in the results of the annual census as shown by higher rates observed after 2015 [7].

Several studies have documented critical periods of excess weight gain in childhood and adolescence [9,16,17]. Such information can be useful in knowing the specific ages for more intense targeting of obesity interventions. The prospective study by Geserick et al. [5], based on data obtained from a German patient registry including children from birth to 19 years of age, showed that the majority of children who developed obesity by age three years remained obese into adolescence. They stated that the most rapid increase occurred during the preschool years in children with both high and normal birth weights. The authors concluded that the rate of weight gain should be closely monitored for all children before the age of six. This was also recommended by the Fit Futures study [18], which determined the degree of tracking of overweight from 2–17 years of age in a cohort of Norwegian children. The authors found that in adolescents with overweight, the prevalence of overweight increased continuously from two years of age, with an important increment between the ages 2–4 and 5–7. The study by Cunningham et al. [19], which analyzed the incidence of obesity among elementary school children in the USA, reported that between the ages of 5 and 14 years, the highest incidence was more likely to have occurred prior to entering kindergarten, and primarily among children who had entered kindergarten with established overweight.

Our study also shows that the emergence of excess weight during this developmental period is linked to various indicators of poverty, as was also reported in a previous Chilean study, which determined the obesity risk from 2009 and 2013 among students attending the first grade. The results showed that although the less vulnerable children (middle-low socio-economic status) had the highest prevalence of obesity. The largest increase in obesity during the period, occurred among the more vulnerable children [20].

The Chilean indigenous population (mainly the Mapuche) has higher poverty levels than the rest of the population and Westernization of lifestyle habits have affected them more strongly. A study by Bustos et al. [21], based on databases of children in first grade from 1997 to 2004, found that the probability of an indigenous child having obesity was 6% higher than for a non-indigenous child.

Low maternal education has also been associated with increased risk of childhood obesity. In Chile, the National Health Survey of 2016 [22] reported the prevalence of obesity in adults to be 10 percentage points higher for those with less than eight years of schooling, compared to those with higher education. These results are also in line with data from other countries. A large European study from 11 countries [23] that included four- to seven-year-old children, found that low maternal education was a significant risk factor for early childhood obesity, with an odds ratio (OR) of 1.58 (95% 1.34, 1.85) in both boys and girls. Interestingly, in contrast to some studies, especially from Europe, in our study, the place of residence was not associated with excessive weight gain. In Chile, children living in rural areas are a minority, have access to the same types of foods, and are as equally sedentary as urban children given the post-transitional stages of the country. Indeed, rurality has been described as a protective factor for obesity in Chilean women but as a risk factor for men [24].

High birth weight has consistently been associated with obesity in childhood and adolescence. In a multinational study, Iscole, which included 5141 children aged 9–11 years, the authors reported that the odds ratios of obesity were significantly higher among children whose birth weight was >3500 g, compared to the reference group with birth weight, 2500–2999 g [25]. The Iscole study showed that the risk of obesity in relation to birth weight was different depending on the country´s income level, with the risk starting to rise after 4000 g in high-income countries. The type of association was different by sex: It was a U-shaped association among boys, while for girls, it was linear. A study including Korean participants aged 12–18 years [26], showed that those born in the highest quartile of birth weight, were more likely to be overweight, this association being more evident in girls, as found in the Iscole study.

In our study, the odds ratio of four-year-old normal-weight children with a high birth weight having overweight or obesity at six years was significantly higher for both boys and girls. In Chile, it has been reported that maternal overweight among women beneficiaries of the public health system reaches 63.6% (half corresponds to obesity); this is a matter of concern for childhood obesity rate given the well-known relationship between maternal obesity and high birth weight [27].

Grandmothers play an important role in children’s upbringing, especially among lower socioeconomic families [28]. Although there is no evidence locally of a possible link between the type of foods consumed and/or activity engaged by children cared for by grandmothers in comparison to those cared by a parent, a recent doctoral thesis based on the Chilean Longitudinal Survey on Early Childhood [29] found that when a child lives with a grandmother in the same house, there is a 20% higher probability that the child is obese, compared to one who lives with one or both parents. This result was the same regardless of socioeconomic level.

Our study has some limitations. It is based on secondary data however its measurements are considered to be valid. This was demonstrated in a study, which examined the concordance between the anthropometric data collected by JUNAEB and INTA on first-grade students in 2009. Results showed that there was no significant difference between the prevalence of obesity calculated with JUNAEB’s data versus INTA’s analysis [30].

We only had a limited amount of biological and behavioral variables to be included as potential predictors of obesity development, and hence could not explore the influence of such factors as maternal obesity, smoking at home, TV time, and dietary and physical activity habits. Previous studies have shown that Chilean children have overconsumption of processed foods and sugar-sweetened beverages, especially milk flavored and fruit juices [31,32]. A recent study, which examined the consumption of these beverages among Chilean preschool children found that the total beverage calories from dairy beverages, substitutes and fruit drinks amounted to around 250 kcal per day [33]. In addition, Chilean children are very sedentary, as shown by two report cards characterizing the country’s physical activity situation of children and adolescents. The most recent report (2018) graded the overall physical activity for the country with a D minus [34].

A major strength of this study is the large population size (483,000 children with anthropometry at both ages) and our ability to test for several socio-demographic effects. Our results can be extrapolated to around 70% of Chilean preschool children that attend public schools. We believe these results are also very relevant to preschool children in other regions of Latin America and the Caribbean as well as countries facing similar stages of the nutrition, demographic and economic transition.

In conclusion, the growth trajectory between preschool and school years has received less attention than the early years. Our study with a large sample of Chilean preschoolers from public schools contributes to the evidence showing that this is an important period for obesity development. The factors that predicted excessive weight gain in this period are mostly related to poverty and, therefore, suggest that prevention efforts should be focused on these higher risk populations.

## Figures and Tables

**Table 1:** Socioeconomic and health-related characteristics of the population of prekindergarten (2011–2015) by sex (percentage).

	Boys (*N* = 232,369) %	Girls (251,140) %
Belongs to an indigenous group	12.4	12.5
Does not attend health check-ups regularly	6.8	7.0
Previous daycare attendance	44.9	44.0
Lives in rural areas	10.8	10.5
Grandmother or other relative in charge of child after school	27.8	28.1
Father with only primary education	22.0	22.7
Mother with only primary education	22.5	22.5
Unemployed father	25.8	25.5
Unemployed mother	43.6	43.5
Attends very vulnerable school	52.0	49.6
No breastfeeding	7.8	7.6
Birth weight ≥ 4000 g	10.8 *	6.8

* χ^2^ significantly higher for boys compared with girls (*p* < 0.05).

**Table 2:** Prevalence of overweight and obesity in boys in prekindergarten (PK) and in the 1st grade (1st G).

Year in PK	% Overweight		% Obesity		BMIZ Mean (SD)	BMIZ Mean (SD)
	In PK	In 1st G, Two Years Later	In PK	In 1st G, Two Years Later	In PK	In 1st G, Two Years Later
2011	28.6	26.6	21.2	26.1	1.1 (1.25)	1.21 (1.32)
2012	27.7	26.8	21.0	25.5	1.1 (1.26)	1.2 (1.31)
2013	28.6	27.0	21.5	24.4	1.12 (1.24)	1.15 (1.3)
2014	27.4	27.1	22.6	24.7	1.14 (1.3)	1.16 (1.31)
2015	27.9	26.4	23.1	24.0	1.15 (1.28)	1.14 (1.31)
Mean for the period 2011–2015	27.9	26.8	22.1	24.8	1.1 (1.1)	1.17 (1.2)

**Table 3:** Prevalence of overweight and obesity in girls in prekindergarten and in 1st grade.

Year in PK	% Overweight		% Obesity		Mean BMIZ	Mean BMIZ
	In PK	In 1st G	In PK	In 1st G	in PK	in 1st grade
2011	27.6	28.6	17.7	20.2	0.95 (1.2)	1.04 (1.2)
2012	27.0	28.5	17.9	20.2	0.95 (1.2)	1.04 (1.18)
2013	28.1	28.9	18.2	19.3	0.97 (1.2)	1.02 (1.17)
2014	27.6	28.5	19.3	19.8	0.99 (1.25)	1.02 (1.17)
2015	27.4	27.7	19.5	19.2	1.01(1.23)	1.01 (1.18)
Mean for the period 2011–2015	27.5	28.4	18.7	19.7	0.98 (1.2)	1.02 (1.1)

**Table 4:** Multivariate logistic regression model showing predictive variables associated with normal-weight prekindergarten children developing overweight or obesity by first grade, by sex.

	Boys	Girls
	OR (95% CI)	OR (95%) CI
Indigenous ethnic group	1.18 (1.11–1.23) **	1.08 (1.02–1.13) *
Mother with primary education	1.07 (1.011–1.13) *	1.19 (1.13–1.25) **
Mother with secondary education	1.04 (0.99–1.09) ^1^	1.09 (1.05–1.14) **
Child cared for by grandmother or other relative after school	1.07 (1.01–1.13) **	1.06 (1.02–1.1) *
Attending a very vulnerable school	1.06 (1.02–1.1) *	1.05 (1.01–1.09) *
Father unemployed	0.95 (0.9–1.00)	0.96 (0.91–1.01)
Father with sporadic job	1.02 (0.97–1.06)	1.05 (1.00–1.09) *
Birth weight ≥ 4000 g	1.46 (1.38–1.55) **	1.47 (1.37–1.57) **

Adjusted by age. Reference categories: Non-indigenous; >12 years of education; child cared for by either parent after school; attending a less vulnerable school (receives less than 75% of free school meals daily); a father with a formal job; birth weight <4000 g. *p* < 0.05 *; *p* < 0.01 **; ^1^ *p* = 0.056; Hosmer–Lemeshow (*p* = 0.91 for boys and 0.32 for girls).
